# Chitosan, the Marine Functional Food, Is a Potent Adsorbent of Humic Acid

**DOI:** 10.3390/md9122488

**Published:** 2011-11-28

**Authors:** Jeen-Kuan Chen, Chao-Hsien Yeh, Lian-Chen Wang, Tzong-Horng Liou, Chia-Rui Shen, Chao-Lin Liu

**Affiliations:** 1 Environment and Biotechnology Department, Refining and Manufacturing Research Institute, CPC Corporation, Chia-Yi 60051, Taiwan; Email: 078450@cpc.com.tw; 2 Department of Chemical Engineering, Ming Chi University of Technology, 84 Gung-Juan Road, Taishan, New Taipei 24301, Taiwan, Email: chyen@mail.mcut.edu.tw (C.-H.Y.); thliou@mail.mcut.edu.tw (T.-H.L.); 3 Department of Parasitology, College of Medicine, Chang Gung University, 259 Wen-Hwa 1st Road, Kweishan, Tao-Yuan 24301, Taiwan; Email: wanglc@mail.cgu.edu.tw; 4 Department of Medical Biotechnology and Laboratory Science, Chang Gung University, 259 Wen-Hwa 1st Road, Kweishan, Tao-Yuan 33302, Taiwan; Email: crshen@mail.cgu.edu.tw; 5 Graduate School of Biochemical Engineering, Ming Chi University of Technology, 84 Gung-Juan Road, Taishan, New Taipei 24301, Taiwan

**Keywords:** chitosan, humic acid, adsorbent, Blackfoot

## Abstract

Chitosan is prepared by the deacetylation of chitin, the second-most abundant biopolymer in nature, and has applicability in the removal of dyes, heavy metals and radioactive waste for pollution control. In weight-reduction remedies, chitosan is used to form hydrogels with lipids and to depress the intestinal absorption of lipids. In this study, an experimental method was implemented to simulate the effect of chitosan on the adsorption of humic acid in the gastrointestinal tract. The adsorption capacity of chitosan was measured by its adsorption isotherm and analyzed using the Langmuir equation. The results showed that 3.3 grams of humic acid was absorbed by 1 gram of chitosan. The adsorption capacity of chitosan was much greater than that of chitin, diethylaminoethyl-cellulose or activated charcoal. Cellulose and carboxymethyl-cellulose, a cellulose derivative with a negative charge, could not adsorb humic acid in the gastrointestinal tract. This result suggests that chitosan entraps humic acid because of its positive charge.

## 1. Introduction

Blackfoot disease (BFD) is an endemic chronic peripheral vascular disease that occurs along the southwest coast of Taiwan [1,2,3]. Patients with similar symptoms were also found in China, The Philippines, India, Romania, Mexico, Chile and Argentina [4,5,6]. The disease results in black discoloration and ulceration of the extremities. After gangrene develops, entire limbs are eventually affected [1,2]. A causal association between BFD and drinking well water has been revealed by an epidemiological study [7]. A fluorescent compound purified from well water and identified as humic acid (HA) was reported to induce BFD-like symptoms in mice [8,9,10,11]. It also shows the HA causes damage to the cell [11,12]. Crystallized HA is stable to heat and acid-base action, and it has been characterized as a polyphenolic-carboxylic polymer containing both carboxyl and hydroxyl groups as the main functional groups [13]. Humic acid is abundant in soil, plants, well water and other water sources and is able to enter the human body in a variety of ways [14].

Chitosan is the fully or partially deacetylated form of chitin, the second most abundant organic polymer on earth next to cellulose [15,16]. Typically, chitosan is produced in shrimp or crab exoskeletons, but it is also obtained from fungal cell walls [17,18]. The structure of chitosan is similar to that of chitin and cellulose, except for the amine group at carbon-2, which is replaced by the *N*-acetyl group in chitin and the hydroxyl group in cellulose. Most commercial and laboratory-created chitosan contain *N*-acetlyglucosamine (NAG) and glucosamine repeating units linked by β-glycosidic bonds [19,20,21]. The degree of deacetylation (DD value) depends on the source or manner of preparation of chitosan; glucosamine units predominate in chitosan, while NAG units are predominant in chitin [22].

A series of experiments have been performed to demonstrate the biomedical function of chitin, chitosan and their derivatives [16,18,21,23,24,25,26,27]. It was shown the hypocholesterolemic properties of chitosan [28,29,30]. The activity was due to the presence of positively charged amine groups on chitosan that could bond with the negative charges on fatty acids or bile acids by ionic interaction, while the triglycerides, cholesterol and sterols were bound due to hydrophobic interactions [31]. In environment and pollution control, chitosan was reported to remove acid dyes from textile wastewater [32,33]. In addition to organic dyes, chitosan has been shown to adsorb cadmium, lead, cooper and hexavalent chromium ions [33,34,35,36]. The amine groups of chitosan serve as a chelating site for transition metal ions, and the adsorption capacity increases with the increase in surface area of chitosan beads [37]. Hence, chitosan is a versatile substance for the removal of harmful compounds from the environment. 

For human and mammalian animals, chitosan is a nontoxic dietary polysaccharide. Although chitosan is known to inhibit the uptake of fat and helps the removal of many chemicals in food via adsorption, chitosan itself is resistant for absorption in gastrointestinal tract [38,39]. In the stomach, the chitosan can be dissolved and remains stable in the acid condition [38,39]. Therefore, the molecules can be adsorbed by chitosan before entering the absorption stage in the intestine. Therefore, chitosan is considered as one of the functional foods to deplete the chemical molecules for promoting the health. We assumed that HA in the food would be removed via the adsorption by chitosan in the stomach. To prove the hypothesis, the current study aims to assess the capacity of chitosan to adsorb HA in the mimic gastrointestinal environment. The mechanism of adsorption is also discussed.

## 2. Experiments

### 2.1. Concentration Analysis of Humic Acid

Raw HA solution isolated from the soil was purchased from Honlih Ltd. Company (Taiwan). HA was precipitated by adding HCl and the supernatant discarded. The sediment was then dissolved in H_2_O and neutralized with NaOH. After removing suspended particle, the filtrate was lyophilized as standard HA sample. HA standard solution used in this study was prepared by dissolving HA in H_2_O. The UV-visible absorption spectrum of HA solutions showed a maximum over the range 190–240 nm. A wavelength of 230 nm was selected to ensure sufficient precision for relatively concentrated HA solutions (30 µg/mL). Measurements for doubling dilutions from stock HA solution at 32 µg/mL were performed in a quartz cell of 1 cm path length at a wavelength of 230 nm by a Hitachi Model U-2000 spectrophotometer. The calibration curve (*r*^2^ = 0.9989) was applied. 

### 2.2. Adsorption Analysis to Mimic Conditions in the Gastrointestinal Tract

The adsorption analysis conducted in this study was similar to that in previous publications [40,41] and is described below. One half gram of cellulose, diethylaminoethyl cellulose (DEAE-cellulose), carboxymethyl cellulose (CM-cellulose), chitin, chitosan or activated charcoal was added to 50 mL of a 0.1 M HCl solution containing HA at a concentration of 40 µg/mL. These solutions of pH 1.0 were shaken at 37 °C for 1 h or the desired period as experimental design in a thermostatically controlled shaker bath and then 70 mL of 0.2 M Na_2_HPO_4_ solution was added by titration within 5 min to maintain a final pH value of 7.1 to 7.4. When sampling, 1 mL of solution was applied and the insoluble matter was removed by a bench-top centrifuge at 10,000 × *g* for 10 min. The supernatant was collected and the concentration of HA was measured by a spectrophotometer of U-2000 from Hitachi.

### 2.3. Preparation of Chitosans with Different Degree of Deacetylation Values

Chitosans with different DD values were prepared from chitin with DD value of 25.0% by alkaline deacetylation as described before. And that is, the deacetylation reaction was performed in 10-fold volume of concentrated NaOH under nitrogen to avoid oxidation. The reaction time was 2 h for chitosans with similar molecular weights [42].

The DD value of chitosan was measured by the first derivative UV-spectrophotometry method (1DUVS) with NAG as the calibration curve [43]. Briefly, the absorption spectra of chitosan in various acetic acid solutions were scanned within 190–250 nm. The zero crossing point (ZCP) was obtained by superimposing 1DUVS of 10, 20, and 30 mM of acetic acid solutions at 203 nm. The DD values of the chitosan samples were determined by the formula:

                                DD＝ 100 − [A/(W − 204A)/161 + A] × 100                                         (1)

where A is the amount of NAG determined and W is the mass of chitosan sample used [43]. 

### 2.4. Batch Experiment for Adsorption Isotherms

The procedure for the adsorption experiments was the same as that described previously in this study except that a fixed amount (0.1 g) of DEAE-cellulose, chitin, chitosan or activated charcoal was used. The aqueous solutions with different HA concentrations were placed in a 0.5 L flask and shaken at 37 °C in a thermostatically controlled shaker bath. After the pH of the solution had been achieved neutrality over a 1-hour period, the residual concentration of HA was measured with standard curve by spectrophotometry.

## 3. Results and Discussion

### 3.1. Adsorption of Humic Acid by Different Adsorbents

**Figure 1:**
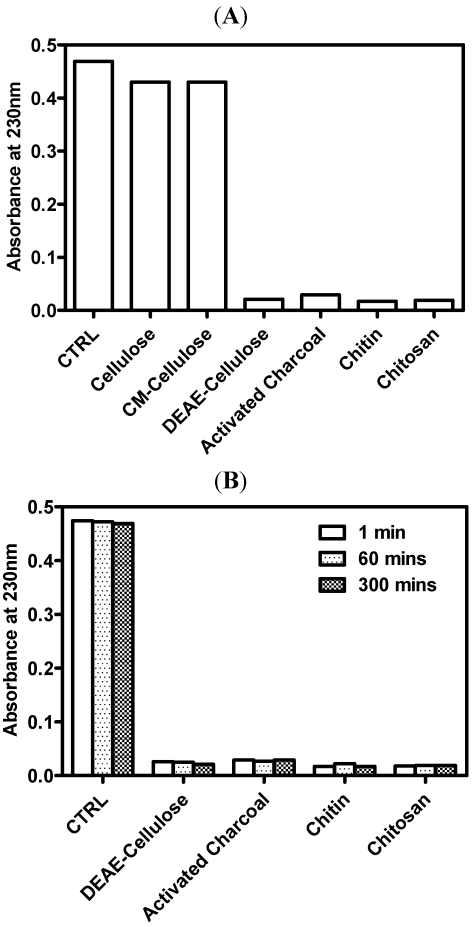
Adsorption of humic acid by different adsorbents. One half gram of cellulose, CM-cellulose, DEAE-cellulose, activated charcoal, chitin, and chitosan was added to 50 mL of HA (40 µg/mL) in 0.1 M HCl. After the incubation at 37 °C for 1 h or the designed period as indicated, the reaction was stopped by the addition of 0.2 M Na_2_HPO_4_. After the centrifugation, the mixture supernatants were measured the concentration of remaining humic acid by spectrophotometer at 230 nm. The representative results were shown (*N* ≥2). CTRL means the control group, in which the humic acid alone without adsorbents was added in the test solution. (**A**) Adsorption capacity of the adsorbents; (**B**) Adsorption rate of the adsorbents.

Chitosan is a linear polymer of predominantly glucosamine units and behaves as a polyelectrolyte at acidic pH values. Because of its ability to form ionic bonds at low pH values, chitosan can bind to different types of anions, such as bile acids or free fatty acids [44]. In the present study, 0.1 M HCl solution was used to imitate gastric acid in the stomach based on a previous study [40]; the pH value of the solution was then raised to 7.1–7.4 to simulate the environment of the upper intestinal tract. Activated charcoal, a well-known adsorbent of organic substances by van der Waal’s forces, was used as a positive control. In their chemical structure, chitosan and cellulose are very similar. In acid condition, only the chitosan is protonized. Therefore, cellulose, cellulose derivatives with charged molecules, DEAE-cellulose of positive charge and CM-cellulose of negative charge, were also investigated. As shown in (Figure 1A), DEAE-cellulose, activated charcoal, chitin and chitosan adsorbed HA effectively. DEAE-cellulose, a cellulose derivative with positive charges, adsorbed HA dramatically, while cellulose and CM-cellulose, both bearing negative charges, did not adsorb HA. This finding suggests that chitosan adsorbs HA by ionic interaction or van der Waal’s forces.

The adsorption rate was monitored (Figure 1B). The result showed that the adsorption capacity of chitosan was similar at different incubation times (1, 60 and 300 min) at neutral pH values, which were not obviously exchanged during the incubation time. These results support the finding of Popa *et al.* [45]. In their study, chitosan and the polyphenols formed a complex, and only 5% of polyphenol was released from the complex after 2 h in a weakly alkaline environment (pH 7.8). Further, the release rate of polyphenols from chitosan-polyphenol complexes was low at neutral pH values, and nearly zero after 90 min.

### 3.2. Adsorption Isotherms

**Figure 2:**
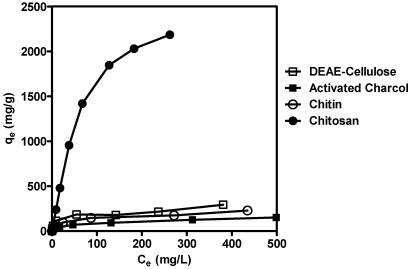
Adsorption isotherms for HA on chitosan, DEAE-cellulose, chitin and activated charcoal at 37 °C. A fixed amount (0.1 g) of DEAE-cellulose, activated charcoal, chitin, and chitosan was added to 50 mL of HA (40 µg/mL) in 0.1 M HCl. After the incubation at 37 °C for 2 h, the reaction was stopped by 0.2 M Na_2_HPO_4_. After the centrifugation, the mixture supernatants were measured the concentration of remaining humic acid by spectrophotometer at 230 nm. The adsorption isotherms, q_e_ as the amount of HA adsorbed per gram of adsorbent and C_e_ as the concentration of HA remaining in the solution, were calculated and the representative results were shown (*N* = 3).

Adsorption isotherms are important to describe how adsorbates will interact with adsorbent. Therefore, these isotherms are critical for optimizing the use of chitosan as an adsorbent [42]. The adsorption isotherms for DEAE-cellulose, chitin, chitosan and activated charcoal were shown in Figure 2. Where q_e_ is the amount of HA adsorbed per gram of adsorbent and C_e_ is the concentration of HA remaining in the solution. The data show that chitosan possesses a much higher adsorption capacity than the other three adsorbents.

It is necessary to analyze the adsorption process by theoretical or empirical equation. The most widely used equation is the Langmuir equation [42], which is represented as:

                                                θ = q_e_/q_∞_ = K C_e_ (1 + K C_e_)                                                 (2)

where θ is the fractional coverage, q_∞_ is the amount of HA adsorbed. Per gram of adsorbent with 100 percent coverage and K is the Langmuir constant.

The essential characteristics of the Langmuir equation can be expressed in terms of the separation factor, RL, which is defined as:

                                                R_L_ = 1/(1 + K C_0_)                                                               (3)

where C_0_ is the highest initial HA concentration (mg/L).

The value of R_L_ indicates that the shape of the isotherm is either unfavorable (R_L_ > 1), linear (R_L_ = 1), favorable (0 < R_L_ < 1) or irreversible (R_L_ = 0). The parameters in the Langmuir equation and separation factors calculated for chitosan, DEAE-cellulose, chitin and activated charcoal are listed in Table 1. Our results indicate that approximately 3.3 grams of HA was adsorbed by 1 gram of chitosan. The adsorption capacity of chitosan was much greater than those of chitin, DEAE-cellulose and activated charcoal. This suggests that the interaction between chitosan and HA is predominantly due to ionic bonding, much like that between chitosan and polyphenolic compounds, as has been reported previously [45].

**Table 1:** Parameters of the Langmuir equation and separation factors for the adsorption of humic acid on different adsorbents obtained at 37 °C.

Sample	K (L/mg)	q_∞ _(mg/g)	R_L_
Chitosan	1.01 × 10^−^^2^	3.33 × 10^3^	4.74 × 10^−3^
DEAE-cellulose	1.21 × 10^−^^1^	2.13 × 10^2^	1.92 × 10^−2^
Chitin	4.54 × 10^−^^2^	1.89 × 10^2^	5.17 × 10^−2^
Active charcoal	2.49 × 10^−^^2^	1.54 × 10^2^	6.26 × 10^−2^

### 3.3. Adsorption Isotherms for Chitosans with Different DD Values

**Figure 3:**
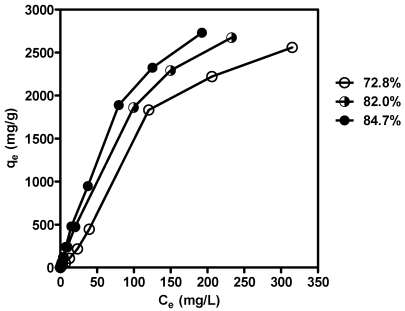
Comparison of adsorption isotherms for humic acid on chitosans with DD values of 72.8%, 82.0% and 84.7% at 37 °C. Chitosans of 0.1 g with different DD values (72.8%, 82.0% and 84.7%, respectively) was added to 50 mL of HA (40 µg/mL) in 0.1 M HCl. After the incubation at 37 °C for 2 h, the reaction was stopped by 0.2 M Na_2_HPO_4_. After the centrifugation, the mixture supernatants were measured the concentration of remaining humic acid by spectrophotometer at 230 nm. The adsorption isotherms, q_e_ as the amount of HA adsorbed per gram of adsorbent and C_e_ as the concentration of HA remaining in the solution, were calculated and the representative results were shown (*N* ≥2).

To confirm the ionic interaction between chitosan and HA, chitosans with different DD values were prepared. The reaction time was controlled for chitosans with similar molecular weights [42]. The DD values for chitosan prepared in 40% NaOH at 110 °C, 50% NaOH at 110 °C and 50% NaOH at 135 °C were calculated to be 72.8%, 82.0% and 84.7%, respectively, by the 1DUVS method. Figure 3 shows the adsorption isotherms for chitosans with different DD values. The adsorption capacity of chitosan on HA increased with increasing DD values. 

According to the results obtained in this study, we suggest that chitosans with a high DD value readily dissolve in the stomach because of the presence of gastric acid. Therefore, their surface area is maximal under these conditions. The dissolved chitosan mixes with HA to form chitosan-HA complexes and subsequently an insoluble gel form in the small intestine. Chitosan’s binding to HA is supported by the positive charges on glucosamine units. This is why chitin and DEAE-cellulose, both with a less positive charge and less surface area, adsorbed less HA than chitosan. These results also interpret the low adsorption capacity of complex beads prepared from activated clay and chitosan, which were prepared by Chang *et al*. [46]. The maximum amount of HA adsorbed was 149 mg per gram of complex bead, which was insoluble in acid condition and possessed less surface area.

Chitosan is a versatile functional food for obesity prevention, hypercholesterolemia inhibition and immune stimulation [38,39,47]. The adsorption of hazardous materials is also conducted in this study. Adsorption of humic acid in the presence of sun-flower seed oil has been performed in another experiment (data not shown). It suggested that adsorption effect and fat entrapping of chitosan could occur synergistically due to the different mechanism. It was reported that chitosan formed a thin layer on the surface of fat droplets to prevent digestion by enzymes [38,40]. As a result, there is enough surface area of chitosan to expose for adsorption of humic acid effectively in the presence of a lipid source. *In vitro* simulation of gastrointestinal condition was conducted using HCl and Na_2_HPO_4_ in this study. However, we have conducted adsorption analysis on HA in this study. This study may not completely imitate the real *in vivo* conditions owing to the complicated dietary composition in the gastrointestinal tract. It is worth conducting further investigation to evaluate the BFD prevention effect of chitosan.

## 4. Conclusions

Several clinical trials have concluded that chitosan is an effective and safe dietary fiber for weight reduction and control of hyperlipidemia. The results of the present study show that chitosan is an effective adsorbent of humic acid in the gastrointestinal tract, and that its adsorption capacity is dependent on the DD value. Chitosan could be used as a novel dietary fiber supplement for the prevention of Blackfoot disease.

## References

[1] Tseng W.P., Chen W.Y., Sung J.L., Chen J.S. (1961). A Clinical Study of Blackfoot Disease in Taiwan, an Endemic Peripheral Vascular Disease. Guoli Taiwan Daxue Yixueyuan Yanjiu Baogao.

[2] Hseu Y.C., Lu F.J., Engelking L.R., Chen C.L., Chen Y.H., Yang H.L. (2000). Humic acid-induced echinocyte transformation in human erythrocytes: Characterization of morphological changes and determination of the mechanism underlying damage. J. Toxicol. Environ. Health A.

[3] Hseu Y.C., Yang H.L. (2002). The effects of humic acid-arsenate complexes on human red blood cells. Environ. Res..

[4] Alaniz S., Armengol J., Leon M., Garcia-Jimenez J., Abad-Campos P. (2009). Analysis of genetic and virulence diversity of cylindrocarpon liriodendri and *C. macrodidymum* associated with black foot disease of grapevine. Mycol. Res..

[5] Petit E., Gubler W.D. (2007). First report of cylindrocarpon liriodendri causing black foot disease of grapevine in California. Plant Dis..

[6] Agustí-Brisach C., Gramaje D., León M., García-Jiménez J., Armengol J. (2011). Evaluation of vineyard weeds as potential hosts of black-foot and petri disease pathogens. Plant Dis..

[7] Yu H.S. (1984). Blackfoot disease and chronic arsenism in southern Taiwan. Int. J. Dermatol..

[8] Lu F.J. (1990). Blackfoot disease: Arsenic or humic acid?. Lancet.

[9] Lu F.J., Liu T.M. (1986). Fluorescent compounds in drinking water of blackfoot disease endemic areas: Animal experimental model. Taiwan Yi Xue Hui Za Zhi.

[10] Lu F.J. (1990). Fluorescent humic substances and blackfoot disease in Taiwan. Appl. Organomet. Chem..

[11] Hseu Y.C., Wang S.Y., Chen H.Y., Lu F.J., Gau R.J., Chang W.C., Liu T.Z., Yang H.L. (2002). Humic acid induces the generation of nitric oxide in human umbilical vein endothelial cells: Stimulation of nitric oxide synthase during cell injury. Free Radic. Biol. Med..

[12] Ting H.C., Yen C.C., Chen W.K., Chang W.H., Chou M.C., Lu F.J. (2010). Humic acid enhances the cytotoxic effects of arsenic trioxide on human cervical cancer cells. Environ. Toxicol. Pharmacol..

[13] Hartenstein R. (1981). Sludge decomposition and stabilization. Science.

[14] Lu F.J., Yamamura Y., Yamauchi H. (1988). Studies on fluorescent compounds in water of a well in blackfoot disease endemic areas in Taiwan: Humic substances. Taiwan Yi Xue Hui Za Zhi.

[15] Gooday G.W. (1990). Physiology of microbial degradation of chitin and chitosan. Biodegradation.

[16] Chen J.K., Shen C.R., Liu C.L. (2010). *N*-Acetylglucosamine: Production and applications. Mar. Drugs.

[17] Mathur N.K., Narang C.K. (1990). Chitin and chitosan, versatile polysaccharides from marine animals. J. Chem. Educ..

[18] Liu C.L., Shen C.R., Hsu F.F., Chen J.K., Wu P.T., Guo S.H., Lee W.C., Yu F.W., Mackey Z.B., Turk J. (2009). Isolation and identification of two novel sds-resistant secreted chitinases from *Aeromonas schubertii*. Biotechnol. Prog..

[19] Shen C.R., Chen Y.S., Yang C.J., Chen J.K., Liu C.L. (2010). Colloid chitin azure is a dispersible, low-cost substrate for chitinase measurements in a sensitive, fast, reproducible assay. J. Biomol. Screen..

[20] Chen J.K., Shen C.R., Yeh C.H., Fang B.S., Huang T.L., Liu C.-L. (2011). *N*-Acetyl glucosamine obtained from chitin by chitin degrading factors in *Chitinbacter tainanesis*. Int. J. Mol. Sci..

[21] Aam B.B., Heggset E.B., Norberg A.L., Sorlie M., Varum K.M., Eijsink V.G. (2010). Production of chitooligosaccharides and their potential applications in medicine. Mar. Drugs.

[22] Singh D.K., Ray A.R. (2000). Biomedical applications of chitin, chitosan, and their derivatives. J. Macromol. Sci. Polym. Rev..

[23] Yang C.J., Liu Y.K., Liu C.L., Shen C.N., Kuo M.L., Su C.C., Tseng C.P., Yen T.C., Shen C.R. (2009). Inhibition of acidic mammalian chitinase by rna interference suppresses ovalbumin-sensitized allergic asthma. Hum. Gene Ther..

[24] Liu Y.K., Yang C.J., Liu C.L., Shen C.R., Shiau L.D. (2010). Using a fed-batch culture strategy to enhance raav production in the baculovirus/insect cell system. J. Biosci. Bioeng..

[25] Shen C.R., Juang J.H., Tsai Z.T., Wu S.T., Tsai F.Y., Wang J.J., Liu C.L., Yen T.C. (2011). Preparation, characterization and application of superparamagnetic iron oxide encapsulated with *N*-[(2-hydroxy-3-trimethylammonium)Propyl] chitosan chloride. Carbohydr. Polym..

[26] Shen C.R., Wu S.T., Tsai Z.T., Wang J.J., Yen T.C., Tsai J.S., Shih M.F., Liu C.L. (2011). Characterization of quaternized chitosan-stabilized iron oxide nanoparticles as a novel potential magnetic resonance imaging contrast agent for cell tracking. Polym. Int..

[27] Hossain S., Rahman A., Kabir Y., Shams A.A., Afros F., Hashimoto M. (2007). Effects of shrimp (*Macrobracium rosenbergii*)-derived chitosan on plasma lipid profile and liver lipid peroxide levels in normo- and hypercholesterolaemic rats. Clin. Exp. Pharmacol. Physiol..

[28] Ebihara K., Schneeman B.O. (1989). Interaction of bile acids, phospholipids, cholesterol and triglyceride with dietary fibers in the small intestine of rats. J. Nutr..

[29] Sugano M., Fujikawa T., Hiratsuji Y., Nakashima K., Fukuda N., Hasegawa Y. (1980). A novel use of chitosan as a hypocholesterolemic agent in rats. Am. J. Clin. Nutr..

[30] Bokura H., Kobayashi S. (2003). Chitosan decreases total cholesterol in women: A randomized, double-blind, placebo-controlled trial. Eur. J. Clin. Nutr..

[31] Colombo P., Sciatto A.M. (1996). Nutritional aspects of chitosan employment in hypocaloric diet. Acta Toxicol. Ther..

[32] Kim C.Y., Choi H.M., Cho H.T. (1997). Effect of deacetylation on sorption of dyes and chromium on chitin. J. Appl. Polym. Sci..

[33] Da Sacco L., Masotti A. (2010). Chitin and chitosan as multipurpose natural polymers for groundwater arsenic removal and As_2_o_3_ delivery in tumor therapy. Mar. Drugs.

[34] Muraleedharan T.R., Venkobachar C. (1990). Mechanism of biosorption of Copper(Ii) by ganoderma iucidum. Biotechnol. Bioeng..

[35] Udaybhaskar P., Iyengar L., Rao A.V.S.P. (1990). Hexavalent chromium interaction with chitosan. J. Appl. Polym. Sci..

[36] Coughlin R.W., Deshaies M.R., Davis E.M. (1990). Chitosan in crab shell wastes purifies electroplating wastewater. Environ. Prog..

[37] Rorrer G.L., Hsien T.Y., Way J.D. (1993). Synthesis of porous-magnetic chitosan beads for removal of cadmium ions from wastewater. Ind. Eng. Chem. Res..

[38] Rodriguez M.S., Albertengo L.E. (2005). Interaction between chitosan and oil under stomach and duodenal digestive chemical conditions. Biosci. Biotechnol. Biochem..

[39] Staffolo M.D., Martino M., Bevilacqua A., Montero M., Rodríguez M.S., Albertengo L. (2011). Chitosan interaction with iron from yoghurt using an *in vitro* digestive model: Comparative study with plant dietary fibers. Int. J. Mol. Sci..

[40] Kanauchi O., Deuchi K., Imasato Y., Shizukuishi M., Kobayashi E. (1995). Mechanism for the inhibition of fat digestion by chitosan and for the synergistic effect of ascorbate. Biosci. Biotechnol. Biochem..

[41] Narayani R., Rao K.P. (1995). Ph-Responsive gelatin microspheres for oral delivery of anticancer drug methotrexate. J. Appl. Polym. Sci..

[42] Juang R.S., Tseng R.L., Wu F.C., Lin S.J. (1996). Use of chitosan in lobster shell wastes for colour removal from aqueous solutions. J. Environ. Sci. Health.

[43] Tan S.C., Khor E., Tan T.K., Wong S.M. (1998). The degree of deacetylation of chitosan: Advocating the first derivative Uv-Spectrophotometry method of determination. Talanta.

[44] Muzzarelli R.A.A. (1996). Chitosan-based dietary foods. Carbohydr. Polym..

[45] Popa M.I., Aelenei N., Popa V.I., Andrei D. (2000). Study of the interactions between polyphenolic compounds and chitosan. React. Funct. Polym..

[46] Chang M.Y., Juang R.S. (2004). Adsorption of tannic acid, humic acid, and dyes from water using the composite of chitosan and activated clay. J. Colloid Interface Sci..

[47] Muzzarelli R.A.A. (2010). Chitins and chitosans as immunoadjuvants and non-allergenic drug carriers. Mar. Drugs.

